# Raman Microspectroscopy as a Tool to Elucidate the Efficacy of Topical Formulations Containing Curcumin

**DOI:** 10.3390/ph12010044

**Published:** 2019-03-23

**Authors:** Ievgeniia Iermak, Ana Paula da Silva, Cristina Kurachi, Vanderlei Salvador Bagnato, Natalia Mayumi Inada

**Affiliations:** 1 São Carlos Institute of Physics, University of São Paulo, Trabalhador Sao-carlense Av., 400, São Carlos, SP 13566-590, Brazil; cristina@ifsc.usp.br (C.K.); vander@ifsc.usp.br (V.S.B); nataliainada@ifsc.usp.br (N.M.I); 2 PDT Pharma Ind. Com. Pharmaceutical Products LTDA, Marechal Deodoro da Fonseca Str., 67, Cravinhos, SP 14140-000, Brazil; ana.paula@pdtpharma.com.br

**Keywords:** Raman microspectroscopy, photodynamic therapy, onychomycosis, curcumin, photosensitizer, drug delivery, topical pharmaceutical formulation

## Abstract

The success of the onychomycosis treatment is directly associated with factors such as the choice of the medication, the administration route, and the pharmaceutical formulation. Photodynamic therapy (PDT) is an emerging and promising technique indicated for onychomycosis treatment. For this application, the main challenge is the efficient delivery of the photosensitizer (PS). Curcumin is widely used as a PS, however it is an unstable molecule and it is a challenge to develop a formulation with good penetration into the nail plate, maintaining the stability of curcumin. In this study, the molecular mechanisms underlying the efficacy of two topical formulations containing curcumin used in a clinical trial for onychomycosis treatment were analyzed by Raman microspectroscopy. It is shown that curcumin is present in both formulations in aggregated and non-aggregated states, and in aggregates it is present in different conformations, depending on the interaction with the solvent. This proves to be critical for efficient and uniform PS delivery to the nail and its complete use during the treatment. These analyses are showing how promising Raman microspectroscopy is in understanding the molecular mechanisms of the efficiency of photosensitizers and are helping to improve the development of pharmaceutical formulations.

## 1. Introduction

Onychomycoses are fungal infections that affect the nail plate, leading to thickening, hardening, discoloration, and disintegration of the nail. It accounts for more than 10% of the worldwide incidence of this disease and represents 30% of all nail infections [1,2]. It can be caused by several fungal species of dermatophyte, non-dermatophyte filamentous fungi (NDFF), and yeast [3]. Currently, it is considered a public health problem due to the high incidence of the disease that can be related to several factors such as hygiene habits, age, sex, social activity, and climate, among others [3]. Diverse topical and systemic antifungal agents can be used for onychomycosis treatment [4,5], however the topical formulations show low efficacy, and oral formulations may cause adverse effects, especially due to their toxic effect on liver and kidneys [6]. Development of the new ways to treat onychomycosis are increasingly relevant due to the growing incidences of resistant microbial species to the available drugs and relatively low efficacy of existing ways treatments. Photodynamic therapy for the treatment of onychomycosis has shown promising results [7,8,9,10,11,12]. In photodynamic therapy (PDT), a photosensitizing compound is activated by light and, in the presence of oxygen, leads to the production of several reactive oxygen species (ROS), inactivating microorganisms [13,14,15], inducing tumor cells death [16,17,18,19] and causing the death of other abnormal cells [20,21,22,23]. The great advantage of the technique for microorganism inactivation is the unlikely possibility of treatment selection of resistant microorganisms to several applications and minimum side effects comparing to systemically used oral drugs. 

Curcumin is a component obtained from Curcuma Longa rhizome and has been studied as a potential photosensitizing compound for the inactivation of microorganisms. In addition, the compound is used for having bactericidal effects on localized surface infections. Initially, curcumin was used primarily in the food industry, but showed various biological effects, such as suppression of carcinogenesis, prevention of the proliferation of a wide variety of tumor cells (skin, lung, stomach, colon, and breast), and anti-inflammatory, antioxidant, and bactericidal activities [24,25,26,27]. Another advantage of curcumin is the low cost of its extraction, which makes curcumin-based pharmaceutical formulations cost-efficient.

Silva and cols [8] reported promising results regarding the use of curcumin solutions for onychomycosis treatment in patients who had unsatisfactory results after conventional therapies and were affected by the disease for more than five years. In the patients with the nail plate affected, the curcumin solution was applied to the nail plate and then irradiated with a light-emitting diode (LED) based device with a wavelength centered at 450 nm [8]. In this previous published study, it has been observed that curcumin in solution does not stay in close contact with the lesion due to the limitation of its standard formulation and the structural features of the nail plate. Thus, the authors are presenting a more complete molecular analysis of two different topical formulations (hydrophilic gel and water/oil cream) containing curcumin, for onychomycosis treatment by the Photodynamic Therapy technique with the aim to comprehend the differences between these two pharmaceutical options that are now presenting significant clinical results [10]. 

Raman microspectroscopy constitutes an elegant tool to study biological and other types of samples containing water and provides insights on molecular mechanisms of the processes occurring in the sample. The Raman spectrum of a molecule is unique, which makes it possible to identify it in a mixture, or in a biological tissue [28]. With confocal Raman microscopy, it is possible to study materials and biological samples without using markers, and the resulting image has information about their chemical composition and conformational state of the molecules in every pixel of the image [29,30,31,32]. Raman microspectroscopy was applied in many studies for pharmaceutical products design, for screening solid forms to find optimal solid forms of the drug [33,34], and for phase transformations [35,36,37] and co-crystal formation [38] to optimize the early stages of drug development. In this study, we analyzed the two formulations with curcumin, mentioned above, and their interaction with the light and the nail plate in order to explain the difference in their clinical PDT efficacy.

## 2. Results

**Figure 1:**
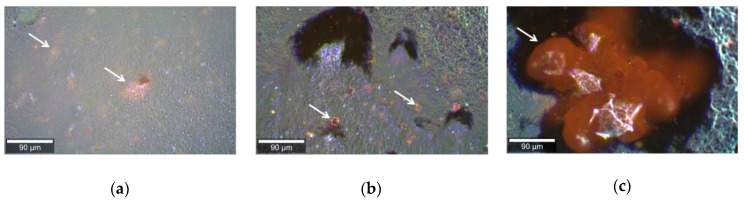
Micrographs of cream (**a**) and gel (**b**,**c**) containing curcumin, showing the presence of small aggregates of curcumin in a cream and both small (**b**) and big (**c**) ones in a gel.

Different pharmaceutic formulations, containing the same concentration of curcumin (1.5% *w*/*w*) were studied using Raman microspectroscopy. In both formulations, curcumin is present in the form of particles, distributed around the base (Figure 1). Additionally, the base itself contains curcumin; however, in lower concentrations than in the particles. The immediate difference between the formulations can be observed from the images shown on Figure 1: The cream contains only relatively small sized particles (Figure 1a), while the gel contains both small (Figure 1b) and large (Figure 1c) pieces of curcumin. 

**Figure 2:**
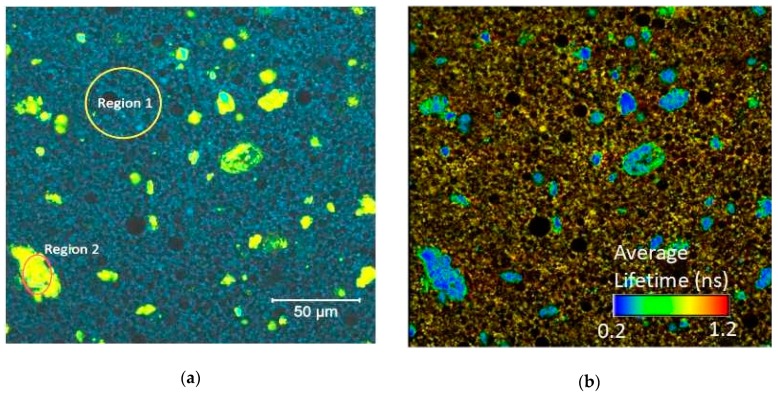

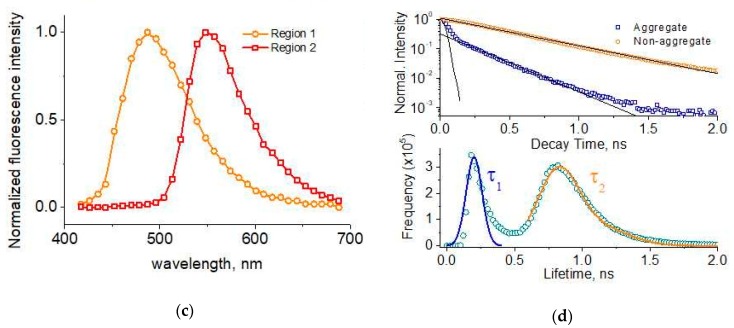
Spectral fluorescence image of cream (**a**) and corresponding fluorescence lifetime imaging microscopy (FLIM) image (**b**). Fluorescence spectra of regions 1 and 2 from (**a**,**c**). Fluorescence decays and average fluorescence lifetimes distribution for the same regions (**d**).

Fluorescence spectral and lifetime imaging measurements were performed with both formulations and show that curcumin in formulations is present both in the base and in the particles and fluorescence spectra, and that the average fluorescence lifetimes of these two forms are different. Figure 2 shows the difference in fluorescence spectra (panels a and c) and average fluorescence lifetimes (panels b and d) between the particles and curcumin distributed in the base for cream. It can be observed that curcumin fluorescence is quenched essentially in the particles (aggregates, region 2 on Figure 2a) and fluorescence emission spectrum of the aggregates is red-shifted comparing to the spectrum of the curcumin distributed in the base of the cream (region 1 on Figure 2a).

However, observed difference in the fluorescence of aggregated and dissolved curcumin is not enough to explain the difference in the PDT and clinical efficacy of the two formulations. To see if there is any difference in the molecular structure of curcumin its Raman spectra and images were recorded for both formulations.

**Figure 3:**
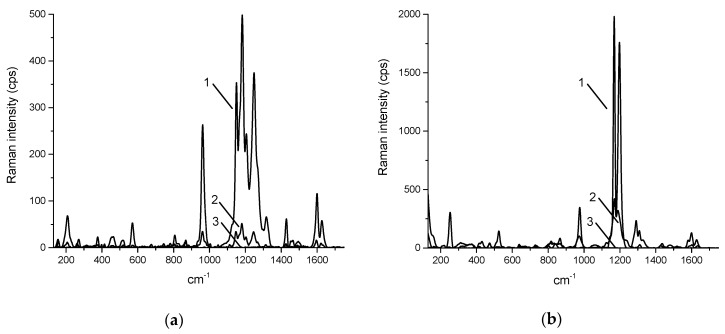
Typical Raman spectra of cream (**a**) and gel (**b**) containing curcumin, where 1—Raman spectrum of aggregated curcumin, 2—Raman spectrum of curcumin, dissolved in the base, and 3—Raman spectrum of the base without curcumin.

Obtained Raman spectra of curcumin within cream and gel revealed substantial differences in the appearance of some bands (for example, 1252 cm^−1^) and their intensity relative to each other (for example, 1183 and 1600 cm^−1^) (Figure 3). It should be noted that even though the base of both cream and gel has Raman spectra, their intensities are weak comparing to the peaks of curcumin, and they contribute to the background, but the intensity of their Raman peaks is too low to contribute to the overall spectra. The bands can be assigned to C–CH stretching (572 cm^−1^), C=O or C–OH stretching (960–963 cm^−1^), enol C–O or C–CH stretching (1149–1153 cm^−1^), enol C–O or CH_3_ stretching (1181–1183 cm^−1^), enol C–O stretching (1199–1206 cm^−1^, 1252 cm^−1^), C–CH stretching (1315–1320 cm^−1^), in plane bending of aromatic (CCC, CCH) (1429–1434 cm^−1^), aromatic C=C stretching (1600–1602 cm^−1^), C=O or C=C stretching (1625–1629 cm^−1^) [39,40,41]. 

One of the differences between the spectra of cream and gel is that in all recorded spectra of the cream the Raman bands of curcumin stay exactly in the same positions, while in the gel they are shifted 2–5 cm^−1^ between different spectra.

**Figure 4:**
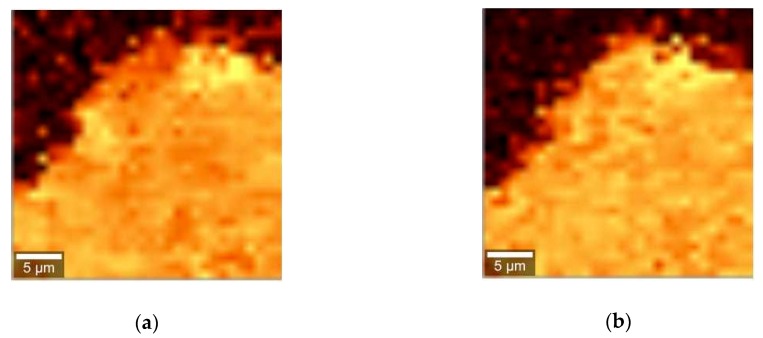

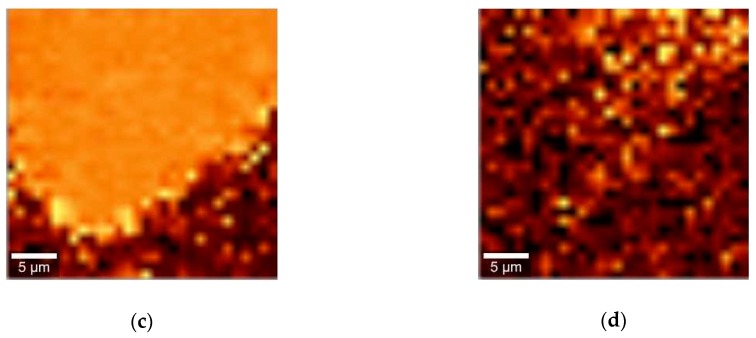
Distribution of Raman peaks at 1182 cm^−1^ and 1252 cm^−1^ in the particles present in the cream ((**a**,**b**), respectively) and gel ((**c**,**d**), respectively), represented in false colors.

Imaging of the particles had shown that the main difference between curcumin spectra when it is dissolved in the cream and in the gel is the absence of the peak at 1251 cm^−1^ in many particles in the gel, meanwhile this peak is always present in the cream. Figure 4 shows the example of distribution of the peak at 1182 cm^−1^, which is always present in curcumin spectra, and of the peak at 1252 cm^−1^, in the particles present in cream (Figure 4a,b) and gel (Figure 4c,d) in false colors. As it can be seen from the images, the distribution of both peaks inside the particle coincide in the case of cream, but in the gel the number of pixels showing presence of the 1252 cm^−1^ peak is much lower than the number of pixels containing the 1182 cm^−1^ peak.

**Figure 5:**
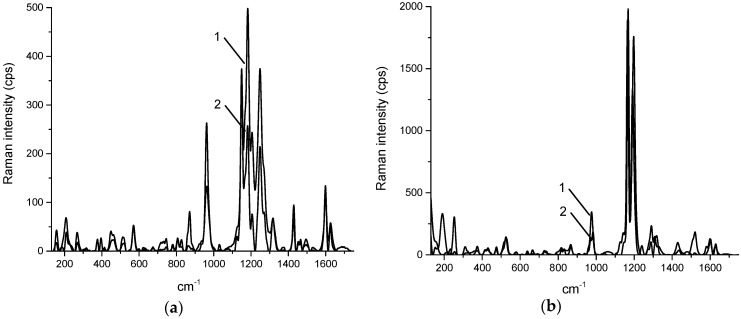

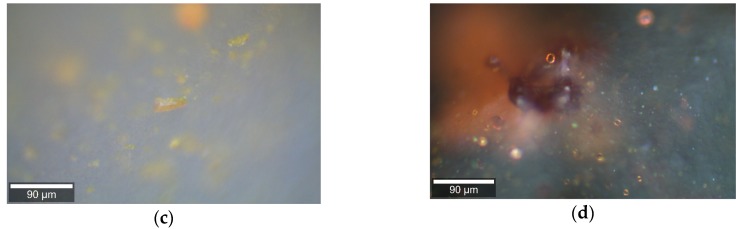
Typical Raman spectra and micrographs of cream ((**a**,**c**), respectively) and gel ((**b**,**d**), respectively) containing curcumin before and after the irradiation in the absence of nail, where 1—Raman spectrum of aggregated curcumin before irradiation, and 2—Raman spectrum of curcumin after irradiation.

Irradiation of the formulations with the blue light of the same wavelength and intensity for the same amount of time as used in PDT treatment in the absence of the nail showed that curcumin is not bleached substantially. Both cream and gel showed presence of the curcumin particles after illumination, and the spectra of the particles remained unchanged (Figure 5).

**Figure 6:**
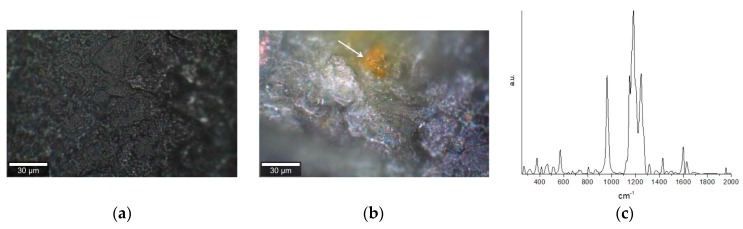
Nail surface after Photodynamic therapy (PDT) treatment with cream (**a**) and gel (**b**). A piece of unbleached curcumin in gel is shown with an arrow in (**b**). (**c**) shows the Raman spectra of the region of the nail containing curcumin from the gel (arrow in (**b**)) after PDT.

PDT with both cream and gel was put on the nail and the Raman spectra were recorded and compared with the control (nail treated with urea, but not treated with any of the curcumin formulations). The nail surface after the PDT with cream appeared to be of even color (Figure 6a) and showed identical Raman spectra in all the parts of the nail plate. However, the nail surface after PDT with the gel showed patterns of two different colors and presence of the non-bleached curcumin particles (Figure 6b—arrow indicates the curcumin particle), confirmed by the Raman spectra (Figure 6c).

In contrast to the gel application on the nail plate, where some spots remained orange in color after PDT treatment and were confirmed to have an unbleached curcumin (Figure 6c), no remnants of curcumin were detected on the nail plate after the PDT treatment with the cream, suggesting that all photosensitizers (PSs) was used during the treatment.

## 3. Discussion

In the present study, we show an important procedure to analyze different formulations for topical administration of curcumin. For better understanding of the results we shall discuss them in two aspects: how our measurements can visualize the differences in the structure of curcumin in different pharmaceutical formulations and how those differences result in a better efficiency of the curcumin in one of the formulations in the tissue during the PDT treatment.

Fluorescent spectral and lifetime imaging shows that curcumin is presented in the gel and cream in two distinct physical states—aggregates and non-aggregates in the base. Recorded Raman spectra showed that in the cream all aggregates contain curcumin in the same conformation, meanwhile in the gel there are two types of aggregates, some of them not showing the peak in Raman spectra at 1252 cm^−1^. According to several studies of curcumin [39,40,41], the Raman peak at 1252 cm^−1^ corresponds to the enol form of the curcumin molecule, which is typically present in solutions, meanwhile keto form is typical for the crystal state of curcumin. This indicates that in the gel not all curcumin is dissolved, and some is still in a form of undissolved crystals. Besides that, we observe that with lower concentration of curcumin in the same gel (0.5%) it does not reach enough saturation for precipitation and, therefore, dominance of enol form in solution is found. This means that higher concentration of curcumin can be dissolved in the cream and will be further available for its delivery to the nail tissue.

These facts indicate the influence of each compound of the cream in the final physical characteristics of the formulation. Besides the spatial physical discrimination, it was observed that molecular structure forms (keto or enol) are presenting in spatial domains differently, as viewed by the Raman spectra. While in the cream (prevalence of non-aggregated molecules) the curcumin is only present in the enol form, and in the gel formulation (with huge presence of aggregates) both molecular structural forms (keto and enol) can be found.

Our observations of both formulations exposed to light indicate that both formulations are stable in the absence of the biological tissue (nail).

In the observation of the two formulations (cream and gel), the main point is to correlate the characteristics observed in the Raman spectra and the efficiency of photosensitizer delivery to the tissue. No heterogeneity and no signature of curcumin was found in the nail plate after the PDT with the cream, meaning that the curcumin in the cream was evenly delivered to the nail plate and all the molecules were available for the PDT. In contrast, presence of unbleached curcumin in the nail after the gel application and the PDT treatment means that the undissolved curcumin in the gel is not delivered efficiently to the nail plate and is excluded from the number of molecules available for PDT. The observation of aggregates of the curcumin molecules after PDT is indicative of a lower efficiency of the gel formulation—aggregates either interact less with oxygen or absorb less light. In both these possibilities the outcome of PDT is compromised.

After the combined analysis (investigating the formulations and their interaction with the target tissue) we are observing strong evidences confirming the findings above in a previous case report published [10] and are now in an extensive ongoing clinical trial (data not shown) when curcumin (1.5% *w*/*w*) formulated in the gel shows lower clinical efficiency than in the cream.

Concerning that enol form of curcumin is stabilized by hydrogen bonding [39], it is within reason to assume that components of the cream are forming hydrogen bonds more readily. In particular, one of the major components (up to 67%) of the cream beeswax are esters [42,43], which are known to be good hydrogen bonds acceptors [44] and can stabilize curcumin in the cream. Another component of the cream, sodium borate, is known to form complexes with curcumin [39], which also should help to dissolve it. Thus, the composition of the cream showed to be better at dissolving curcumin and to delivering it to the nail plate.

As observed in our study, understanding the behavior of the active molecule within its vehicle directs for a better clinical outcome in PDT and could help the pharmaceutical companies to improve the choice of adequate constituents for a better formulation, also maintaining the efficacy and safety of the active substance.

## 4. Materials and Methods

### 4.1. Materials

Cream and gel containing 1.5% (*w*/*w*) of curcumin (Sigma–Aldrich, St. Louis, MO, USA) were prepared by PDT Pharma (Cravinhos, São Paulo, Brazil). Gel composition was ethylenediaminetetraacetic acid (EDTA), glicerol, paraben solution, ethanol 70%, carbomer 940, AMP-95, and pH 7.0. Cream composition was sodium borate, paraben solution, water, beeswax, liquid petrolatum, butil hydroxytoluene, and pH 7.0.

### 4.2. Raman Microspectroscopy

Raman measurements were performed on the WITec Alpha 300 RAS microscope (WITec, Ulm, Germany). The excitation wavelength was 785 nm, detection range was 100–3200 cm^−1^. The images and spectra were collected with 20 × and 50 × magnification objectives (Zeiss, Jena, Germany). Spectra were recorded with the integration time of 10 s and 10 accumulations for cream and gel, and with 20 s and 20 accumulations for nail samples. Images of 30 × 30 μm size were recorded with the integration time of 1 s per spectrum and the spatial resolution of 30 × 30 pixels. Obtained images were processed using WITec ProjectFOUR software, and spectra were processed using WITec ProjectFOUR and Origin 2016 software.

### 4.3. Fluorescence Lifetime Imaging Microscopy

Fluorescence lifetime imaging microscopy (FLIM) measurements were performed with the confocal fluorescent microscope Zeiss-LSM780 (Zeiss, Jena, Germany) using continuous emission diode 405 nm laser and Ar+ 488 nm laser pumping Ti:Sa femtosecond laser (Chameleon XR, Coherent Inc., Santa Clara, Canada) with the pulse width of 100 fs emitting light at 800 nm. Images were collected in the spectral intervals of 400–500 nm (channel 1) and 500–700 nm (channel 2). Obtained images were processed using SPCImage software (Becker & Hickl GmbH, Berlin, Germany).

### 4.4. Photodynamic Therapy

Nail plate cuts were donated by volunteers and the samples were used for the comparison of gel and cream containing curcumin according to the protocol described in [8]. In brief, the nail plate cuts were first treated with urea for one hour to increase penetration of the curcumin formulation into the nail plate. After that, urea was removed and gel or cream containing curcumin was placed on the nail. After 30 minutes the excess of it was removed and a blue light (450 nm LED, 100 mW/cm^2^ as described in [8]) was applied for 20 min. Raman spectra were recorded after the gel and cream application and after the PDT treatment. Nail cuts treated with urea only were used to record the control spectra.
